# Cognitive Control in Opioid Dependence and Methadone Maintenance Treatment

**DOI:** 10.1371/journal.pone.0094589

**Published:** 2014-04-11

**Authors:** Ding-Lieh Liao, Cheng-Yi Huang, Sien Hu, Su-Chen Fang, Chi-Shin Wu, Wei-Ti Chen, Tony Szu-Hsien Lee, Pau-Chung Chen, Chiang-shan R. Li

**Affiliations:** 1 Department of Addiction Psychiatry, Bali Psychiatric Center, Department of Health, New Taipei City, Taiwan; 2 Institute of Occupational Medicine and Industrial Hygiene, College of Public Health, National Taiwan University, Taipei, Taiwan; 3 Department of Psychiatry, Yale University School of Medicine, New Haven, Connecticut, United States of America; 4 Department of Nursing, Oriental Institute of Technology, New Taipei City, Taiwan; 5 Department of Psychiatry, Far Eastern Memorial Hospital, New Taipei City, Taiwan; 6 School of Nursing, Yale University, New Haven, Connecticut, United States of America; 7 Department of Health Promotion and Health Education, National Taiwan Normal University, Taipei, Taiwan; University of Chicago, United States of America

## Abstract

**Background:**

Substance misuse is associated with cognitive dysfunction. We used a stop signal task to examine deficits in cognitive control in individuals with opioid dependence (OD). We examined how response inhibition and post-error slowing are compromised and whether methadone maintenance treatment (MMT), abstinence duration, and psychiatric comorbidity are related to these measures in individuals with OD.

**Methods:**

Two-hundred-and-sixty-four men with OD who were incarcerated at a detention center and abstinent for up to 2 months (n = 108) or at a correctional facility and abstinent for approximately 6 months (n = 156), 65 OD men under MMT at a psychiatric clinic, and 64 age and education matched healthy control (HC) participants were assessed. We computed the stop signal reaction time (SSRT) to index the capacity of response inhibition and post-error slowing (PES) to represent error-related behavioral adjustment, as in our previous work. We examined group effects with analyses of variance and covariance analyses, followed by planned comparisons. Specifically, we compared OD and HC participants to examine the effects of opioid dependence and MMT and compared OD sub-groups to examine the effects of abstinence duration and psychiatric comorbidity.

**Results:**

The SSRT was significantly prolonged in OD but not MMT individuals, as compared to HC. The extent of post-error slowing diminished in OD and MMT, as compared to HC (trend; p = 0.061), and there was no difference between the OD and MMT groups. Individuals in longer abstinence were no less impaired in these measures. Furthermore, these results remained when psychiatric comorbidities including misuse of other substances were accounted for.

**Conclusions:**

Methadone treatment appears to be associated with relatively intact cognitive control in opioid dependent individuals. MMT may facilitate public health by augmenting cognitive control and thereby mitigating risky behaviors in heroin addicts.

## Introduction

Cognitive control is critical to behavioral learning and adaptation in a constantly changing environment. Cognitive control involves response inhibition, error detection, and post-error behavioral adjustment and these processes are compromised in individuals with addiction [1], [2]. Opioid use disorders are associated with impairment in cognitive and affective functions, as demonstrated by neuropsychological assessment alone [3]–[15] (see also van Holst [16] for a review) or in combination with brain imaging [17]–[23].

Studies showed that methadone or buprenorphine maintenance ameliorates some of the cognitive deficits in opiate users [24]–[28]. However, some demonstrated more impairment in individuals on methadone maintenance treatment (MMT), as compared to abstinent heroin users [29], [30] (see also Wang et al. [31] for a review). A recent meta-analysis suggests that MMT is associated with impaired cognitive function across multiple domains [31]. A number of factors, such as experimental setting and behavioral tests as well as sample characteristics, may contribute to this inconsistency. In particular, comorbidity including misuse of other substances and duration of abstinence may affect cerebral structure and cognitive functioning [7], [32]–[34]. For instance, after approximately 26 weeks of abstinence, opioid dependent individuals did not appear to differ from healthy controls in attention and working memory [7]. Previous findings were also at odds as to whether current cocaine dependence contributes to cognitive dysfunction in methadone maintenance patients [35], [36].

Here, we aimed to address the effects of MMT on cognitive dysfunction in individuals with opioid dependence. We examined cognitive control in a large cohort of opioid dependent individuals by using the stop signal task, a behavioral paradigm that has been widely used to study impulsivity in health and disease including substance use disorders [37]–[41] (see Li and Sinha, [2] for a review). Briefly, in the stop signal task, participants respond to a frequent “go” signal and are required to interrupt this habitual response when an infrequent “stop” signal appears. With the difficulty of the stopping process adjusted trial by trial, we are able to characterize the component processes of cognitive control under various computational frameworks [42]–[44]. Specifically, we examined two outcome measures – stop signal reaction time (SSRT) and post-error slowing (PES). The SSRT represents the time required for one to successfully inhibit a prepotent response; a short SSRT thus indicates a better capacity of response inhibition. The PES represents the difference in reaction time when go trials are preceded by a stop error versus another go trial, and is thought to reflect performance monitoring [2], [40]. We compared SSRT and PES between three groups of opioid dependent individuals who were each recently abstinent (up to 2 months), abstinent for 6 months, and under MMT, and an age- and education- matched group of healthy participants.

Thus, by assaying cognitive control with a widely validated behavioral paradigm in a large sample of opioid dependent and healthy control individuals, we address the effects of MMT, abstinence duration, and psychiatric comorbidity on a critical cognitive function. We hope that the results would shed light on the utility of MMT for this chronic, relapsing disorder and the various clinical characteristics that may impact the effects of MMT.

## Methods

### 1. Subjects: clinical characteristics and assessment

The study was conducted according to a protocol (IRB970609-03) approved by the Institutional Review Board of the Bali Psychiatric Center. Individuals with heroin dependence were recruited from two Department of Justice correction agencies in northern Taiwan – the Taipei Detention Center and Sindian Drug Abuser Rehabilitation Center – and the Bali Psychiatric Center. Written informed consent was obtained from all participants after they were given a detailed explanation of the purpose and procedures of the study. When obtaining consents, we emphasized that the study was not mandatory and that refusal to participate would not jeopardize their treatment or legal rights.

Inmates arrested for illicit drug use for the first time were incarcerated at the Taipei Detention Center for a short-term detoxification treatment (≤2 months). Those who were recidivists were transferred to the Sindian Drug Abuser Rehabilitation Center for rehabilitation in a program that included abstention-counseling, group psychotherapy and occupational therapy, for a period of approximately 6 months. Participants in these two groups were thus abstinent each for less than 2 months (opioid dependent and abstinent for a short duration or OD-short; n = 108) and 6 months (OD-long; n = 156). No psychotropic medications were given to participants in the OD-short or OD-long groups. To ensure abstinence, urine toxicology tests were performed every two weeks and after family visits, as well as randomly in the detention centers as part of the treatment and education protocol. A third group of individuals with OD were recruited from the Bali Psychiatric center; they had participated in methadone maintenance treatment program for a period of approximately 6 months at the time of the current study (MMT; n = 65). According to the MMT guidelines in Taiwan, methadone was started at 30 mg/day and individually titrated as needed. The individuals were on stable methadone dosage for at least one month before enrollment. The maintenance dosage ranged from 30 to 60 mg/day. On the day of study, they received psychiatric and drug-use related assessment as well as their daily dosage of methadone before being tested on the stop signal task.

Non-drug using individuals (n = 64) were recruited from the community to participate in the study as healthy controls (HC). HC, OD-short, OD-long, and MMT were group matched in age and years of education. Only men were recruited for the current study because these correction agencies accepted only male inmates.

Board-certified psychiatrists (DLL, CSW and CYH) evaluated all participants for psychiatric illness using the Mini- International Neuropsychiatric Interview [45] and use of other substances. Individuals in the OD-short, OD-long and MMT group all met the criteria for opioid dependence (Diagnostic and Statistical Manual of Mental Disorders, 4^th^ edition, American Psychiatric Association). Exclusion criteria included current or past psychotic disorders, neurological illnesses, or head injuries. Furthermore, participants in the OD-short and OD-long group all tested negative for HIV, because individuals who tested positive for HIV would be transferred to a different detention center, where the staff was specifically trained for the care of the HIV infected patients. MMT participants also received tests for HIV as required by the Center of Disease Control of Taiwan. All MMT participants were HIV negative, verified by lab data in a registry database. In Taiwan, individuals who tested positive for HIV were treated at specialized infective disease clinics under subsidy by the government.

### 2. Behavioral task and outcome measures

Behavioral testing was conducted in an office where it was quiet and free of interruptions. We employed a simple reaction time (RT) task in this stop-signal paradigm, as described in our earlier work [46], [47]. There were two trial types: “go” and “stop”, randomized in presentation. A small dot appeared on the computer screen to engage attention at the beginning of a go trial. After a randomized time interval between 1 and 2 s (the fore-period, uniform distribution), the dot turned into a circle (the “go” signal, approximately 2° visual angle), instructing the subjects to quickly press a mouse button. The circle vanished at button press or after 1 s had elapsed, whichever came first, and the trial terminated. Approximately two thirds of all trials were go trials. The remaining were stop trials. The same small dot appeared on the computer screen to begin a stop trial. The dot was replaced by the go signal following a fore-period, and an additional “X”, the “stop” signal, appeared after the go signal, instructing subjects to withhold button press. Likewise, a trial terminated at button press or when 1 s had elapsed since the appearance of the stop signal. The time interval between the stop and go signals – the stop-signal delay (SSD) – started at 200 ms and varied from one stop trial to the next according to a staircase procedure, increasing and decreasing by 64 ms each following a stop success and error trial.

Subjects were instructed to respond to the go signal quickly while keeping in mind that a stop signal could come up occasionally. Each participant had a practice session prior to the experiment to ensure that they fully understood the task. Because of trial randomization and difference in response speed, actual trial number varied slightly across participants. There were approximately 360 (240 go and 120 stop) trials in an experiment. With the staircase procedure we anticipated that the subjects would succeed in withholding their response in approximately half of the stop trials.

Two outcome measures were obtained. First, we computed the stop signal reaction time (SSRT) to represent the capacity of response inhibition. Specifically, with the staircase procedure, a “critical” SSD was computed that represents the time delay required for a subject to succeed in withholding the response in half of the stop trials [48]. To compute the critical SSD, SSDs across stop trials were grouped into runs (sequences of trials), with each run defined as a monotonically increasing or decreasing series. We derived a mid-run estimate by taking the middle SSD of every second run. The critical SSD was computed by taking the mean of all mid-run SSDs. It was reported that, except for experiments with a small number of trials (less than 30), the mid-run estimate was close to the maximum likelihood estimate of X50 (50% SS in the SST, [49]. Based on the horse race model, the SSRT – the time required for one to stop button press after onset of the stop signal – is then estimated by subtracting the “critical” SSD from the median RT of the go trials [50].

Second, we computed post-error slowing (PES) to index the extent of behavioral adjustment after participants committed an error. As in any reaction time (RT) tasks, RT typically increased following an error in the stop signal task [51], [52]. Thus, we computed the extent of post-error slowing (PES) by subtracting the mean RT of go trials that were preceded by another go trial from the mean RT of go trials that were preceded by a stop error trial.

### 3. Data analyses

We examined differences in psychiatric comorbidity between individuals who underwent MMT and those who did not (OD-short and OD-long combined; i.e., OD-combined) and between OD-short and OD-long groups, using chi-square tests.

For each of the two outcome measures (SSRT and PES) on the stop signal task, we first addressed the effects of opioid dependence and methadone maintenance treatment (MMT) by conducting a one-way analysis of variance to examine group (OD-combined, MMT, and HC) effect, followed by post-hoc planned comparisons. We then examined the effects of abstinence duration by conducting a one-way analysis of variance to examine group (OD-short, OD-long, and MMT) effect, followed by post-hoc planned comparisons. In a covariance analysis, we accounted for the effects of psychiatric comorbidity by including methamphetamine use disorder, alcohol use disorder, nicotine use disorder, depression, anxiety, antisocial personality disorder, and conduct problem as categorical covariates (1 = yes; 0 = no). In the same model, we also examined the main effects of each of the covariates. In addition to *P* values, we reported the effect size or partial Eta squared (*η*p^2^) to show the proportion of the variance in the dependent variable that can be attributed to the effect.

In reporting the results, we accounted for multiple comparisons and considered a *P* value of 0.05/number of comparisons as significant and a *P* = 0.05 as showing a trend toward statistical significance, where the issue of multiple comparisons applies.

## Results

### 1. Clinical characteristics

We compared the prevalence rate of psychiatric comorbidities between MMT and non-MMT (OD-short and OD-long or OD-combined) participants (**Table 1**). Pearson Chi-Square test showed a significant difference between MMT and OD-combined for nicotine use (χ^2^ = 10.332, p = .001) and a trend difference for amphetamine use disorder (χ^2^ = 4.020, *P* = .045), but not alcohol use (χ^2^ = .208, *P* = .649), mood (χ^2^ = 2.238, *P* = .135), anxiety (χ^2^ = .295, *P* = .587), or antisocial personality (χ^2^ = 1.075, *P* = .300) disorder, or conduct problem (χ^2^ = 1.157, *P* = .282). Compared to OD-combined, the MMT group showed a higher proportion of participants with nicotine and amphetamine (trend only) use disorders.

**Table 1:** Demographics and clinical characteristics participants.

Group	Control	Opioid Dependent			
	HC	MMT	OD-		
	(n = 64)	(n = 65)	combined		
			(n = 264)		
				OD-long	OD-short
				(n = 156)	(n = 108)
Age (years)	36.8±10.9	40.2±9.5	36.4±8.5	36.6±8.1	36.0±9.1
Education (years)	9.3±1.9	8.6±2.1	9.2±2.1	9.4±1.7	8.9±2.7
Heroin use duration (years)	0	14.3±8.9	7.2±6.4	7.8±6.4	6.4±6.5
Amph use disorder (%)	0	64.2#	49.0#	55.9‡	38.4‡
Alcohol use disorder (%)	27.3*	60.4	57.0	57.2	56.6
Nicotine use disorder (%)	29.5	86.8#	64.1#	68.4‡	57.6‡
Mood disorder (%)	0	31.5	21.3	31.0	13.2
Anxiety disorder (%)	0	8.6	12.7	17.1	8.8
ASPD (%)	0	5.7	2.0	1.6	2.2
Conduct problem (%)∧	0	62.3	54.2	67.1	34.3

Abbreviations: Amph: amphetamine; ASPD: antisocial personality disorder.

# *P*<.05 between MMT and OD-combined groups.

‡ *P*<.05 between OD-long and OD-short subgroups.

*binge drinking (≥5 drinks over one occasion in the prior month; none met criteria of alcohol abuse or dependence).

∧ self-reported “problems” with law authority including previous jail time for non-drug related violations.

We also compared the OD-short and OD-long groups (**Table 1**). Pearson Chi-Square test showed a significant difference between OD-short and OD-long for amphetamine (χ^2^ = 11.404, *P* = .003) and nicotine use disorder (χ^2^ = 13.577, *P* = .001), as well as conduct problem (χ^2^ = 27.223, *P* = .000), but not for alcohol use (χ^2^ = .219, *P* = .896), mood (χ^2^ = 4.286, *P* = .117), anxiety (χ^2^ = .964, *P* = .617), or antisocial personality (χ^2^ = 1.229, *P* = .541) disorder. The OD-long group showed a higher proportion of participants with amphetamine and nicotine use disorders, and conduct problem, as compared to the OD-short group.

### 2. Stop signal reaction time and post-error slowing: opioid dependence and MMT

Stop signal task performance is summarized in **Table 2**. Participants scored over 95% of the go trials and approximately half of the stop trials, suggesting the success of the tracking procedure. In the below, we examined the two main outcome measures of cognitive control: stop signal reaction time (SSRT) and post-error slowing (PES), which were defined in the Methods.

**Table 2:** Stop signal performance.

Group	Control	Opioid Dependent			
	HC	MMT	OD-		
	(n = 64)	(n = 65)	combined		
			(n = 264)		
				OD-long	OD-short
				(n = 156)	(n = 108)
Go response rate (%)	96.8±1.8	96.7±1.8	96.5±1.6	96.4±1.6	96.6±1.7
median Go RT (ms)	615±138	609±121	704±101	721±83	676±119
Stop error rate (%)	52.1±2.5	52.2±2.4	52.6±1.9	52.7±1.9	52.5±2.0
SSRT (ms)	215±59†	217±57# ‡	325±79† #	343±63‡	298±94‡
PES (ms)	55±50†	26±78	28±61†	28±57	27±68

Abbreviations: RT: reaction time; SSRT: stop signal reaction time; PES: post-error slowing.

† *P*<.05 between HC and OD-combined groups (post-hoc Tukey tests).

# *P*<.05 between MMT and OD-combined groups (post-hoc Tukey tests).

‡ *P*<.05 between MMT vs. OD-long, MMT vs. OD-short, and OD-long vs. OD-short subgroups (post-hoc Tukey tests).

An omnibus analysis of variance (ANOVA) showed that there was a significant difference in SSRT among the three (MMT, OD-combined, and HC) groups (F = 75.290, *P*<.001, *η*p^2^ = .304). Post hoc Tukey tests showed a significant difference between OD-combined and MMT (107.9±11.2 ms, *P*<.001) and between OD-combined and HC (109.9±12.1 ms, *P*<.001). The difference between MMT and HC was not significant (2.3±15.1 ms, *P* = .990). Thus, compared to HC and MMT, OD-combined showed prolonged SSRT or impaired response inhibition.

An omnibus ANOVA showed that there was also a significant group difference in PES (F = 3.731, *P* = .025, *η*p^2^ = .021). Tukey tests showed a significant difference between OD-combined and HC (−27.4±10.3 ms, *P* = .022) and a trend difference between MMT and HC (−29.2±12.8 ms, p = .061) but not between MMT and OD-combined (−1.8±9.5 ms, *P* = .980). Thus, compared to HC, OD-combined showed diminished PES or impaired performance monitoring and post-error behavioral adjustment.

### 3. SSRT and PES: abstinence duration and psychiatric comorbidity

To examine the effects of abstinence duration, we conducted additional ANOVA's on OD-short, OD-long, and MMT. The omnibus ANOVA showed that there was a significant difference in SSRT among groups (*F* = 58.403, *P*<.001, *η*p^2^ = .280). Post hoc Tukey tests showed that all pair-wise comparisons were significant (MMT vs. OD-short: −80.6±12.5 ms, *P*<.001; MMT vs. OD-long: −125.7±11.7 ms, *P*<.001; OD-short vs. OD-long: −45.1±9.5 ms, *P*<.001). Thus, both OD-short and OD-long showed prolonged SSRT – indicative of impaired response inhibition – as compared to MMT, and OD-long was more impaired as compared to OD-short.

The omnibus ANOVA showed that there was no significant difference in PES among the three groups (*F* = 0.20, *P* = .984, *η*p^2^ = .000). Tukey tests similarly did not show a difference between groups (MMT vs. OD-short: −1.5±11.0 ms, *P* = .990; MMT vs. OD-long: −2.0±10.3 ms, *P* = .979; OD-short vs. OD-long: −.59±8.4 ms, *P* = .997).

An ANCOVA for SSRT with group (MMT, OD-short, and OD-long) as the between-subject factor and amphetamine use disorder, alcohol use disorder, and nicotine use disorder, mood disorder, anxiety disorder, antisocial personality disorder, and conduct problem as covariates revealed a significant main effect of Group (*F* = 12.653, *P*<.000, *η*p^2^ = .334), confirming the group main effect. The covariate of amphetamine use disorder was significant (*F* = 6.515, *P* = .011, *η*p^2^ = .028) but not other covariates, including alcohol use (*F* = 1.232, *P* = .268, *η*p^2^ = .005), nicotine use (*F* = 2.173, *P* = .142, *η*p^2^ = .009), anxiety (*F* = 1.423, *P* = .234, *η*p^2^ = .006), mood (*F* = 1.975, *P* = .161, *η*p^2^ = .009), antisocial personality (*F* = .876, *P* = .350, *η*p^2^ = .004) disorder, or conduct problem (*F* = .118, *P* = .732, *η*p^2^ = .001). Thus, the difference in SSRT among MMT, OD-short, and OD-long remained significant when psychiatric comorbidities were taken into account. Furthermore, amphetamine use was associated with a difference in SSRT across groups, an issue that required further analysis (Section 3.4).

An ANCOVA for PES with group (MMT, OD-short, and OD-long) as the between-subject factor and amphetamine use disorder, alcohol use disorder, nicotine use disorder, mood disorder, anxiety disorder, antisocial personality disorder, and conduct problem as covariates revealed no significant results in the main effect of group or covariates: group (*F* = .775, *P* = .640, *η*p^2^ = .030); amphetamine use disorder (*F* = .403, *P* = .526, *η*p^2^ = .002); alcohol use disorder (*F* = .045, *P* = .833, *η*p^2^ = .000); nicotine use disorder (*F* = 1.075, *P* = .301, *η*p^2^ = .005); anxiety disorder (*F* = .583, *P* = .446, *η*p^2^ = .003); mood disorder (*F* = .879, *P* = .349, *η*p^2^ = .004); anti-social personality disorder (*F* = .245, *P* = .621, *η*p^2^ = .001); and conduct problem (*F* = 2.199, *p* = .139, *η*p^2^ = .010).

### 4. SSRT: the effect of amphetamine use

The ANCOVA, as described in Section 3.3, demonstrated a significant effect for amphetamine use on SSRT. We examined the effects of amphetamine use on SSRT, separately for each group. A two-sample t-test showed that there was no difference between amphetamine users and nonusers in the MMT group (*t* = −.417, *P* = .684, equal variance not assumed). There was a significant difference between amphetamine users and nonusers in the OD-short group, with users demonstrating a shorter SSRT, compared to non-users (257±79 vs. 324±94 ms; *t* = −3.799, *P*<.001, equal variance not assumed). There was no difference between amphetamine users and nonusers in the OD-long group (*t* = −.574, *P* = .567, equal variance not assumed).

Thus, compared to nonusers, amphetamine users showed shorter SSRT in the OD-short but not OD-long or MMT group. That is, for participants in the OD-short group, comorbid amphetamine use appears to be associated with less impairment in response inhibition.

### 5. SSRT and PES: the effects of Go RT

We used the median rather than mean GoRT in the computation of SSRT in order to account for the skewness of RT distribution typical of a RT task. In an additional analysis, we included GoRT (in addition to clinical variables, as described in Section 3) as a covariate in the ANCOVA's. The results showed that the main group effect of SSRT remained significant both with MMT, OD-combined, and HC as between-group factors (*F* = 46.558, *p* = .000, *η*p^2^ = .216) and with MMT, OD-short, and OD-long as between-group factors (*F* = 32.934, *p* = .000, *η*p^2^ = .184). Thus, the between-group differences in SSRT remained significant when GoRT was accounted for in covariance analyses.

## Discussion

### 1. Opioid dependent individuals under methadone maintenance treatment appears relatively intact in cognitive control as assessed by the stop signal task

Individuals with opioid dependence under methadone maintenance treatment (MMT) are less compromised in cognitive control. The stop signal reaction time (SSRT) was significantly shorter in the MMT, as compared to the OD-combined group (who did not receive MMT), and did not differ from non-drug using control participants. Post-error slowing was decreased in OD-combined, as compared to HC, and showed a trend toward decrease (*P* = 0.061) when compared to the MMT group. Although a causal relationship cannot be drawn from these cross-sectional findings, these results suggest that at least a critical aspect of cognitive control – response inhibition – as indexed by the SSRT, may potentially be remediated with methadone treatment. This is to be considered along with recent work demonstrating that, as compared to healthy individuals, OD individuals under MMT are associated with structural and functional changes of the brain [19], [53]–[55]. That is, the putative structural and functional brain changes do not appear to hamper OD individuals from improvement in cognitive control under MMT. Thus, these results support earlier work that indicates the importance of MMT in augmenting cognitive control, which would facilitate abstinence and improving quality of life in opioid dependent individuals [56], [57]. By enhancing cognitive control, MMT may also reduce risky behaviors that jeopardize individual and public health [58], [59] and “reactive” thinking that contributes to criminal offenses [60], [61]. Notably, the MMT group has been dependent on heroin for a longer duration (averaged 14.3 years of use), as compared to those who did not receive MMT (averaged 7.2 years of use, Table 1), suggesting that duration of use should not be considered as a factor that discourages the implementation of MMT.

### 2. The effects of abstinence duration on cognitive control

Compared to OD-short participants (abstinent for up to 2 months), participants in the OD-long group (abstinent for approximately 6 months) showed more severe impairment in cognitive control, as indexed by longer SSRT. This difference remained significant even when psychiatric comorbidities were accounted for. This finding suggests that abstinence for 6 months did not appear to help these participants recover from deficits of cognitive control, in contrast with an earlier work [7]. One possible explanation is that the current cohort represented a unique population because participants were recruited from detention centers with many reporting non-drug related legal violations. Thus, one cannot rule out the effects of other potential, unmeasured confounds. For instance, having been detained for a longer period may have made the OD-long group unmotivated and uncooperative, jeopardizing their task performance. Furthermore, over half of the participants reported using one or more of other illicit substances; although psychiatric comorbidities did not seem to contribute directly to these cognitive deficits, we cannot rule out the possibility that these comorbidities interact to compromise cognitive functioning. Likewise, although we included conduct problem as a covariate in data analyses, the nature of conduct problem was not assessed in detail and may vary across the three OD groups. For instance, it is likely that the OD-long group may have committed violence-related crimes and thus represents a population distinct from the OD-short or MMT group. With these considerations, one should be cautious in concluding that prolonged abstinence alone does not appear to be as effective as MMT in ameliorating deficits of cognitive control in opioid dependent individuals. On the other hand, it is notable that MMT is associated with improved cognitive control in chronic opioid addicts who misused alcohol and other illicit substances and were slightly (though not significantly) older than other OD participants [43], [62].

### 3. The effects of comorbid amphetamine use on cognitive control

Many users of heroin or other opioid substances are engaged in polysubstance misuse, as evidenced by the current findings; more than half of the current cohort is comorbid with amphetamine, alcohol, and nicotine use disorders, all of which implicate deficits in cognitive control [41], [63]–[65]. It is generally conceived that use of multiple substances would aggravate cognitive dysfunction as the effects of each substance are likely be to additive or multiplicative. However, empirical studies are few and the findings are not consistent. For instance, earlier studies showed that comorbid use of cocaine abuse may or may not result in additional cognitive deficits in heroin users [35], [36]. Here, we found that, in the recently abstinent/initial offender group (but not in the other two groups), concurrent use of amphetamine appears to diminish the change in SSRT, a finding that cannot be easily reconciled with these earlier reports. Indeed, substances interact to exert their neuropharmacological effects, and more studies are required to systematically investigate this issue. Furthermore, we cannot rule out the possibility that individuals with concurrent amphetamine use and those without differ in critical subject characteristics that might impact cognitive control but were not captured in our assessment.

### 4. Limitations of the study and conclusions

A few additional limitations of the study need to be considered. First, chronic human immunodeficiency virus (HIV) infection is known to occur in higher frequency in intravenous drug users. Chronic HIV infection is known to be associated with cognitive dysfunction [66]. On the other hand, our cohort comprised only of HIV negative individuals; thus, it is not clear whether the current results could be generalized to a broader population of heroin users, who often have comorbid HIV infection. Second, our cohort comprised only of men. Because of gender differences in cognitive and affective control [46], [67]–[69], these findings should be considered specific to men with opioid dependence. Third, we used the stop signal task to assess response inhibition and post-error slowing. Future studies are warranted to address other dimensions of cognitive control, such as rule-based shifting of the mental set. With these considerations, we concluded that MMT improves cognitive control in opioid dependent men.

To conclude, we demonstrated in a large sample of opioid dependent and healthy individuals that methadone treatment is associated with relatively intact response inhibition and post-error slowing in a stop signal task. Longitudinal studies are warranted to evaluate the role of these component processes of cognitive control in treatment retention, relapse prevention, and quality of life in opioid dependent individuals.
